# Real-Time Analysis of *Drosophila* Post-Embryonic Haemocyte Behaviour

**DOI:** 10.1371/journal.pone.0028783

**Published:** 2012-01-05

**Authors:** Christopher J. Sampson, Michael J. Williams

**Affiliations:** Institute of Biological and Environmental Sciences, University of Aberdeen, Aberdeen, United Kingdom; Alexander Flemming Biomedical Sciences Research Center, Greece

## Abstract

**Background:**

The larval stage of the model organism *Drosophila* is frequently used to study host-pathogen interactions. During embryogenesis the cellular arm of the immune response, consisting of macrophage-like cells known as plasmatocytes, is extremely motile and functions to phagocytise pathogens and apoptotic bodies, as well as produce extracellular matrix. The cellular branch of the larval (post-embryonic) innate immune system consists of three cell types—plasmatocytes, crystal cells and lamellocytes—which are involved in the phagocytosis, encapsulation and melanisation of invading pathogens. Post-embryonic haemocyte motility is poorly understood thus further characterisation is required, for the purpose of standardisation.

**Methodology:**

In order to examine post-embryonic haemocyte cytoskeletal dynamics or migration, the most commonly used system is *in vitro* cell lines. The current study employs an *ex vivo* system (an adaptation of *in vitro* cell incubation using primary cells), in which primary larval or pre-pupal haemocytes are isolated for short term analysis, in order to discover various aspects of their behaviour during events requiring cytoskeleton dynamics.

**Significance:**

The *ex vivo* method allows for real-time analysis and manipulation of primary post-embryonic haemocytes. This technique was used to characterise, and potentially standardised, larval and pre-pupal haemocyte cytoskeleton dynamics, assayed on different extracellular matrices. Using this method it was determined that, while larval haemocytes are unable to migrate, haemocytes recovered from pre-pupae are capable of migration.

## Introduction

*Drosophila melanogaster* is a robust model organism, with each life stage providing novel aspects for research into the cellular innate immune response. *Drosophila* embryogenesis is a useful tool for determining the mechanistic factors behind immune cell migration. During early embryogenesis haemocytes migrate out of the head mesoderm, along specific paths such as the ventral nerve cord, eventually populating the entire embryo [1], [2]. *Drosophila* embryos provide an *in vivo* platform in which the powerful genetic tools of *Drosophila* can be utilised to manipulate various proteins and pathways, such as Rho-family small GTPases, in order to elucidate the mechanisms controlling cell migration within an organism [3], [4]. Characterisation of haemocyte motility within the embryo showed that haemocytes can be considered a ‘jack-of-all-trades’ whereby they are implicated in general embryonic development, cellular innate immunity and wound healing, which is similar to an inflammation response [5], [6]. The ability to visualise embryonic haemocytes *in vivo* has meant that latter life stages of *Drosophila* have been overshadowed.

*Drosophila* larval immunosurveillance cells consist of three haemocyte types – plasmatocytes, crystal cells and lamellocytes. Within larvae there are three main populations of haemocytes, those in circulation, those located in the lymph gland and a sub-epidermal sessile-haemocyte population [7]. Haemocyte differentiation occurs in both the lymph gland and sub-epidermal sessile sites. Specification of cell lineages begins with prohaemocytes, where it is proposed that prohaemocytes differentiate into either plasmatocytes and crystal cells, under normal conditions, and plasmatocytes can differentiate into lamellocytes when larvae are attacked by a parasitoid wasp [4], [7]. The various haemocyte cell types are produced during the second wave of haematopoiesis, also known as larval haematopoiesis [8]–[10].

Plasmatocytes, the most abundant haemocyte in circulation in healthy larvae, are rounded in morphology, ranging in size from ∼20–35 µm in diameter, and are the *Drosophila* equivalent to phagocytes. Plasmatocytes are capable of engulfing bacteria and fungi, and are involved in the primary stages of encapsulation against the endoparasitoid wasp *Leptopilina boulardi* [8], [9], [11]. Crystal cells are the second most abundant circulating haemocyte type in healthy larvae. They are similar in size to plasmatocytes and are believed to contain crystals of the zymogen prophenoloxidase. They are responsible for local application of prophenoloxidase, resulting in the local deposition of melanin [12]–[14]. Crystal cells have been widely associated with coagulation and the innate immune response, in particular with encapsulation of parasitoid wasp eggs. Lastly, lamellocytes are the largest haemocyte cell type ranging in diameter from ∼60–250 µm, and have an extremely spread confirmation. They are very rarely observed in healthy larvae, but their numbers increase significantly in parasitized larvae. Lamellocytes main responsibility, known so far, is forming a secondary layer of haemocytes during the encapsulation response [8], [9], [11].

So far *Drosophila* larvae have provided a basic characterisation of haemocyte actin-cytoskeletal dynamics within an immune context [10], [15]–[17]. Still, further information is required to augment this initial characterisation to include a primary standardised data set on their motile behaviour at the larval instar and pre-pupal, stages The novel *ex vivo* method described in this paper allows researchers to bridge that gap between *in vitro* and *in vivo*, by re-creating a system of incubation, which can support primary *Drosophila* haemocytes isolated from larvae or pre-pupae. It must be mentioned, since only circulating haemocytes were tested using the *ex vivo* method, it is possible that the other, lymph gland or sessile, haemocyte populations have distinct properties that cannot be assessed using this method.

Using this technique we have analyzed haemocyte random motility, and provided an initial characterisation of pre-pupal haemocyte motile behaviour in the absence of the Rho family GTPases – Cdc42, Rac1 and Rac2.

## Results

### The different life stages

**Figure 1:**
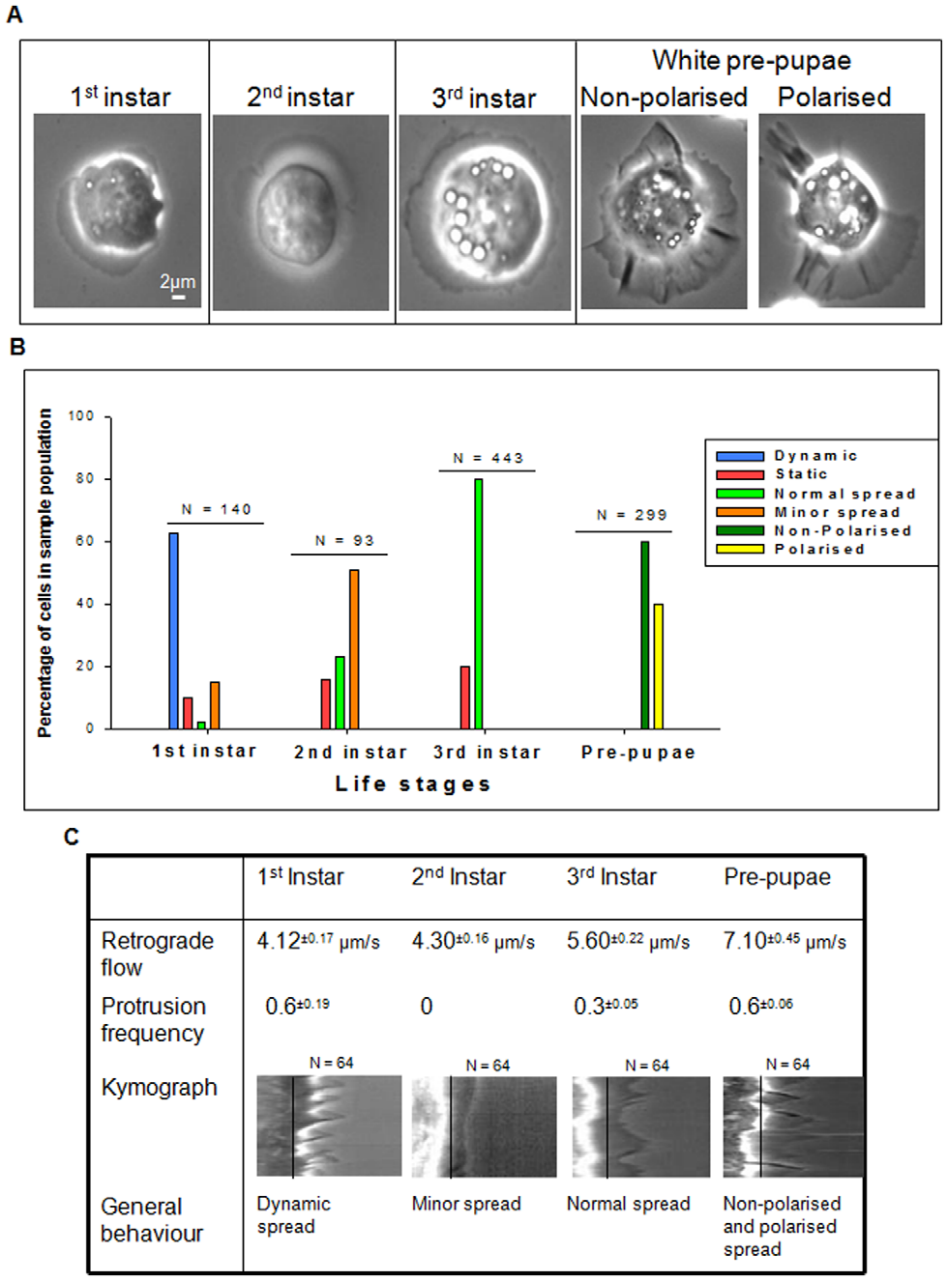
Haemocytes from larval to pre-pupal stages. (A) Figure contains phase contrast images of haemocytes from larval and pre-pupal life stages. In each instance, the captured cell shown proved to best represent the haemocytes at that particular life stage. (B) Haemocytes, from each life stage, were separated based upon there behaviour and morphology. Six classifications were determined by observing the most common attributes between the haemocytes from each life stage. This allowed cells to be placed into either, dynamic, static, normal spread, minor spread, non-polarised, and polarised cellular phenotypes. (C) Kymographs of haemocytes from each life stage were analysed to determine their retrograde flow, protrusion frequency, and general behaviour. The black line across each kymograph implies the base of each protrusion within the plasma-membrane region.

An analysis of haemocyte behaviour at each larval instar was conducted in order to determine whether there was any difference in their centroid and plasma-membrane morphology, and protrusion activity (based on retrograde flow analysis and protrusion frequency analysis). For this investigation, haemocytes were isolated from each larval stage, under *ex vivo* conditions, and analysed by real-time microscopy on gelatine (collagen digest). The general structure of haemocytes from each life stage was the same; they all possessed a centroid region surrounded by a spread plasma-membrane region (Figure 1A). Haemocytes from first instar larvae possessed an actively protruding and retracting plasma-membrane. The centroid region (nucleus and cytoplasm) itself appeared quite active and was visually observed to re-orientate, or rotate in the same direction as the plasma-membrane, in response to reorientation of the plasma-membrane when it was changing the direction of protrusion; this phenotype was labelled as a dynamic. Approximately 60% of the first instar haemocyte population produced a dynamic phenotype (Figure 1B). This was also supported by the presence of a 0.6 protrusion frequency (Figure 1C). The behaviour of haemocytes from second instar larvae was not significantly different from first instar haemocytes (Figure 1A). Although, the behaviour of the centroid region, and plasma-membrane, was less active and more static in comparison to the actively re-orientating centroid regions of first instar haemocytes. The protrusion frequency was 0, the lowest protrusion frequency produced in comparison to all the other life stages. This was labelled as a static phenotype, and it was determined that ∼50% of second instar haemocytes produced this phenotype (Figure 1B).

Haemocytes from third-instar larvae contained a centroid region with multiple vesicular bodies that appeared above the plane of view. These vesicular structures didn't seem to be present in first and second larval instar haemocytes (Figure 1A). The general behaviour of third instar haemocytes was described as normal, meaning the haemocytes were fully adhered and spread, and the plasma-membrane was protruding and retracting, but in a uniform manner. Of the third instar haemocyte population analysed, 80% produced a normal spread phenotype (Figure 1B).

At the pre-pupal life stage, haemocytes appeared in two forms: (1) non-polarised, where cells were adhered and spread, and the plasma-membrane was extremely active, producing multiple membrane ruffles, and (2) polarised, where cells adhered and spread but the plasma-membrane formed a lamellipodium (leading edge) and a trailing edge (Figure 1A). In general, these cells demonstrated greater activity, as shown by the retrograde flow and protrusion frequency, in comparison to larval haemocytes (Figure 1C). Non-polarised haemocytes appeared non-motile, and fixed in placed, but their plasma-membrane continually changed its orientation (Film S1). Polarised haemocytes, on the other hand, were capable of re-distributing the plasma-membrane to form a leading edge and create a trailing edge at the posterior end (Film S2). The migration of these cells was observed as smooth and continuous, similar to neutrophil cell migration [18], [19]. Positioning of the leading edge and trailing edge were not entirely constant, and was actively re-distributed when the cell was making a turn during its path of migration (Film S2).

### Reaction of third instar haemocytes on different ECM substrates

**Figure 2:**
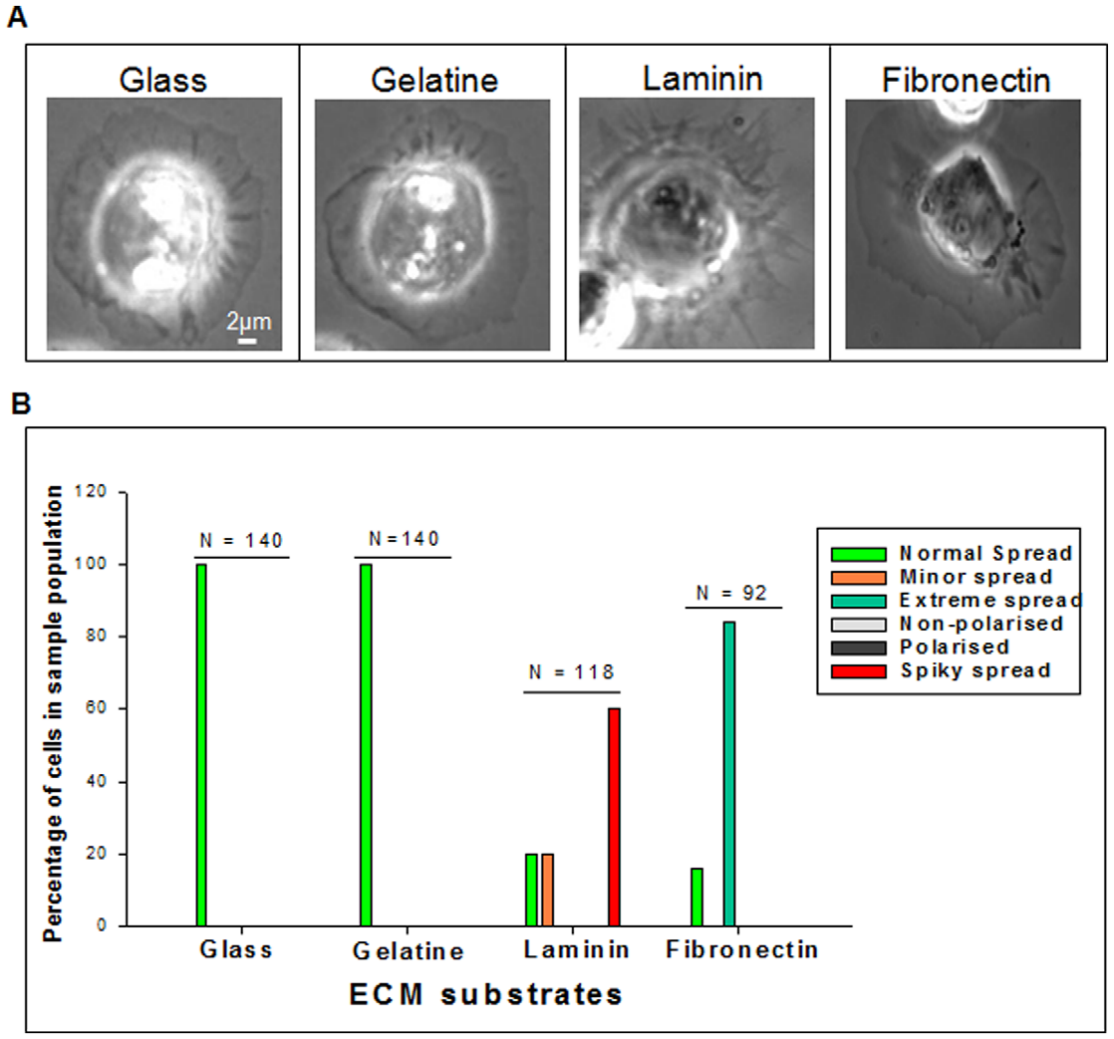
Morphology of third instar haemocytes on various extra-cellular matrices. (A) Adhering and spreading ability of third instar haemocytes on glass, gelatine, laminin and fibronectin. The captured cell provided, is the best representation of general haemocyte behaviour on each respective surface. (B) Each haemocyte was assigned a phenotype, which were created from commonly observed attributes between haemocytes from each surface for spreading. Haemocytes on glass were controls and produced a spread labelled as normal spread. Overall there were six phenotypes, normal spread, minor spread, extreme spread, non-polarised, polarised and spiky spread.

Haemocyte adhesion, spreading, and behaviour were analysed on three different ECM substrates, gelatine, laminin and fibronectin. Controls were produced from third instar haemocytes adhering and spreading over a glass surface. Control cells had uniformly spread plasma-membranes (classed as normal spread) which contained various phase dense ‘notches’ immediately surrounding the centroid region. Controls also possessed a stereotypical lamellipodium that was non-specific in its distribution, under these conditions there appeared to be no filopodia present (Figure 2A).

On the gelatine (collagen digest) substrate, haemocytes from third-instar larvae adhered and spread similarly to controls. The cells maintained a uniform plasma-membrane spreading from the centroid region. General cell morphology was similar to controls, with the presence of a lamellipodium, whilst lacking filopodia (Figure 2A). From the sample population 100% of the cells formed the normal spread phenotype (Figure 2B). On laminin, haemocytes adhered and spread, but the plasma-membrane produced a spiky distribution, forming multiple extensions. There was a definitive centroid region, surrounded by a slightly uniform plasma-membrane, which then formed multiple filopodial-like extensions; approximately 60% of the cells within the sample population formed this phenotype (Figure 2B). Finally, on fibronectin, the haemocytes also adhered and spread. The plasma-membrane was extremely spread and particularly phase sparse, almost transparent in appearance (Figure 2A). From the sample population 84% of the cells formed this phenotype (Figure 2B). Since fibronectin is not a naturally occurring ECM in *Drosophila*, it cannot be concluded if this is a true behaviour of *Drosophila* haemocytes.

### Pre-pupal haemocytes on gelatine

**Figure 3:**
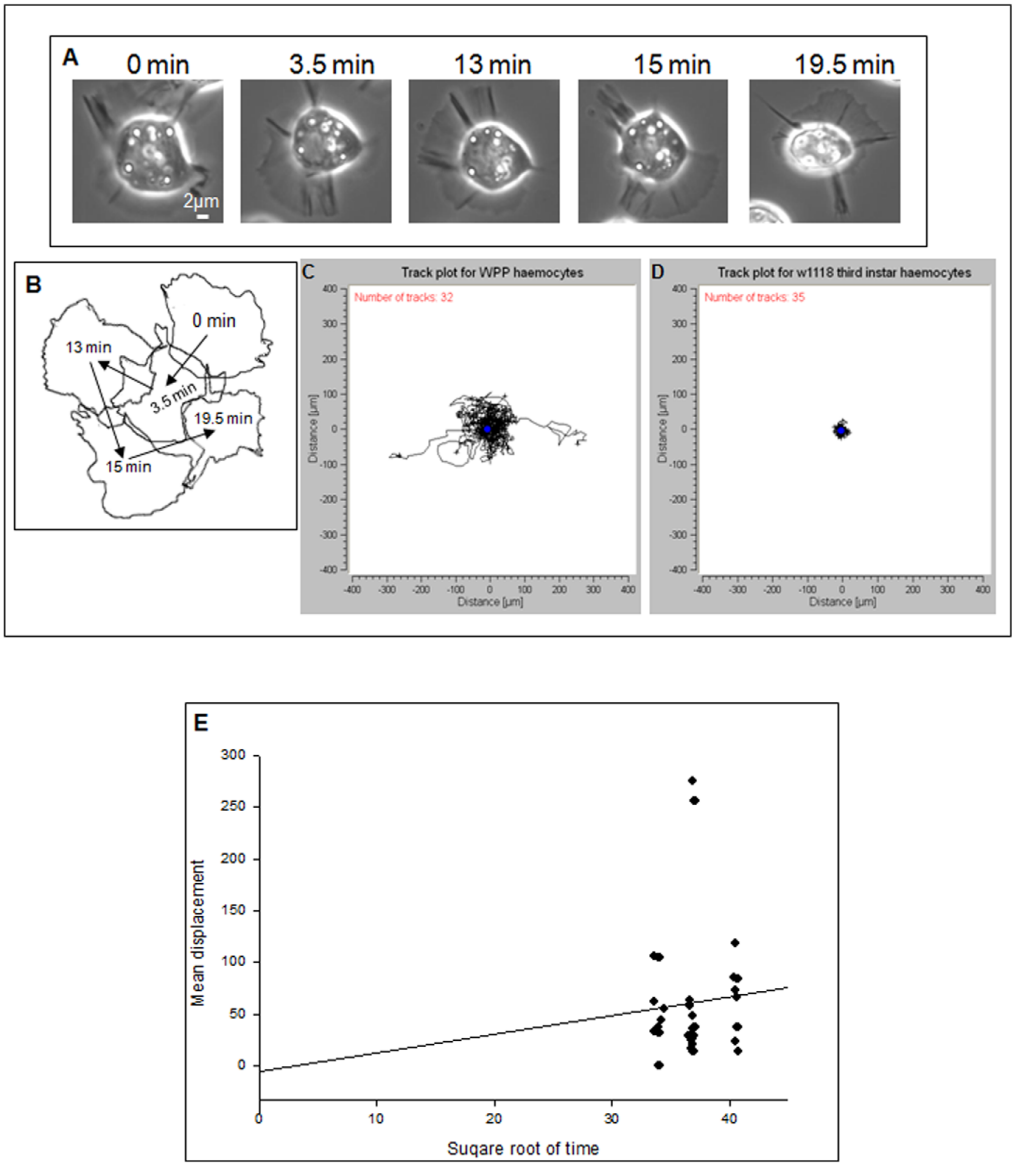
Motility characteristics of haemocytes from white pre-pupae life stage, on a 2D gelatine matrix. Haemocytes from pre-pupae were isolated and incubated in *ex vivo* on a 0.2% gelatine substrate. (A) Haemocytes were observed during real-time imaging under 63× magnification. The collage is of a single migrating haemocyte and the images are of crucial points in that cells journey, and the time at which these cellular events occurred are provided. (B) An outline of a representative cell at each respective time point, the arrows are used to show the vector which the cell took within its path of migration. (C) and (D) Cumulative track plots of a minimum of 30 haemocytes from white pre-pupae and third instar life stages. These of figures show the lack of motility within the third-instar larvae, in comparison to white prepupae (WPP). (E) Displacement of each migrating cell's path, from the WPP track plot. This displacement was plotted against the square root of the total time taken for the each cell to complete its journey. Figure was created in Sigmaplot.

Preliminary analysis of pre-pupal haemocytes determined that they exhibit motile behaviour (Figure 3). From the outset, it was observed that these haemocytes were extremely active, evident through their excessive spreading and higher rates of protrusion and retraction (Film S1 and S2, Figure 1C). Figure 3A and 3B are still images and outlines of each still image, respectively, taken from each step when the cell produced a polarised phenotype. This collage shows that a single cell is capable of multiple points of polarisation within single random journey.

Thirty-two pre-pupal haemocytes were selected at random to create track plots (Figure 3C) and were compared to track plots from third instar haemocytes (Figure 3D). This comparison showed that pre-pupal haemocytes were not only more active than haemocytes from larval stages, but also capable of migrating randomly. It was observed that white pre-pupal (WPP) haemocytes were capable of producing a total path length (total displacement), within a single journey, ranging from 100–350 µm (SE ±10.8). As a comparison, analysis of 35 third instar haemocytes showed no motility and these haemocytes were generally spinning around a central axis up to a maximum distance of 30 µm, which was significantly lower when compared to pre-pupal haemocytes, supported by a two sample Student's T-test, which showed significance with P<0.001.

Pre-pupal haemocytes were observed to migrate in random directions, this was standardised in Table 1 as a summary of some basic characteristics of motile behaviour. Migration reached velocities ranging between 0.25–0.73 µm/s (0.43 µm/s±0.024, (Mean±SE)), with an arrestment coefficient between 1.13–1.50 µm/s (1.25 µm/s±0.020). Such a high arrestment coefficient was supportive of their random migrating behaviour, in that cells stopped migrating regularly to make turns, ranging between 4–7 turns at angles between 0° and 180°. A confinement ratio, ranging between 0–0.30 (0.10±0.014 (Mean±SE)) (arbitrary units) was also determined, suggesting that these cells occupy a small spatial distribution around the original point of adhesion, in random conditions. Finally, the directionality ranged between 0–0.28 (0.11±0.014 (Mean±SE)) (arbitrary units), suggesting that there was no directional migration within this random system and that the cells were capable of auto-polarisation.

**Table 1:** Motile characteristics exhibited by pre-pupal haemocytes in *ex vivo*.

Motility characteristic:	Range:	Mean:	S.E. (±):
Velocity (µm/s)	0.25–0.73	0.43	0.024
Arrestment coefficient (µm/s)	1.13–1.50	1.25	0.02
Confinement ratio	0–0.278	0.1	0.014
Angle of turn (°)	0–180	140	N/A
Number of turns	02-Jul	4	0.173
Directionality	0–0.28	0.11	0.014

Summary of data used to characterise the motility of haemocytes from pre-pupae in 2D, random conditions. A basic characterisation was provided by analysing each motile haemocyte for its migration velocity, arrestment coefficient, confinement ratio, angle of turn, number of turns, and the directionality. The table provides minimal and maximal data points, for a given range, and also an average for each characteristic.

Directed haemocyte migration was also tested and demonstrated *ex vivo* (Film S3). Healthy WPP haemocytes were extracted and incubated *ex vivo* prior to addition of a dissected section of decaying gut, which was separately incubated and then added to the healthy system. It was determined that motile cell behaviour was observed in the direction of the dying tissue debris exuding from the dissected section of gut (Film S3). This was not immediately observed until approximately 40 minutes post addition of the dying tissue and consequent debris. The cells produced long, thin protrusion all over the cell, where contact with the debris initiated movement of the cells toward that target and eventual binding.

### Analysis of haemocyte motility in Cdc42, Rac1, or Rac2 loss-of-function mutants

**Figure 4:**
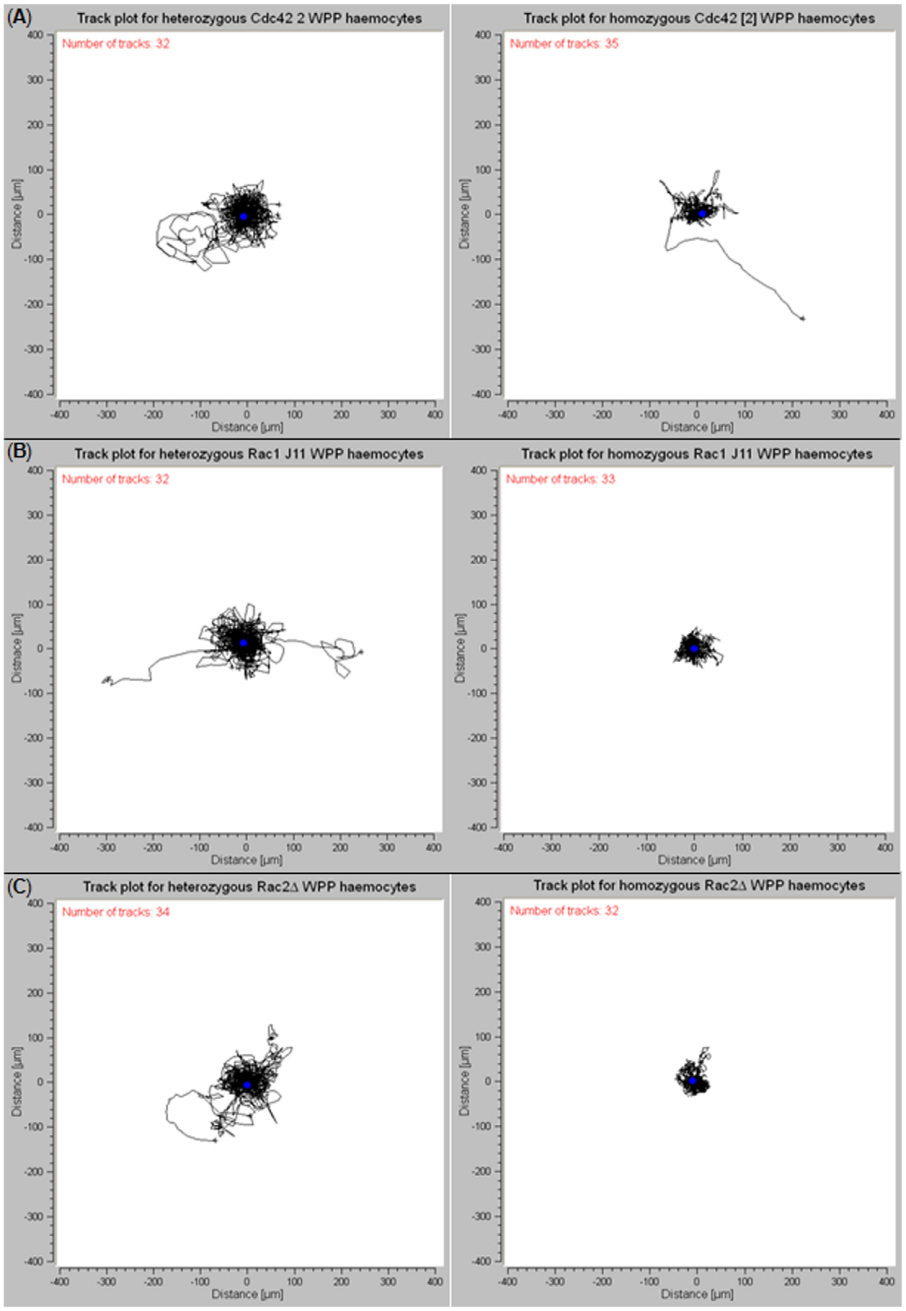
Track plots, for loss-of-function Cdc42, Rac1 and Rac2 white pre-pupae haemocytes. WPP Haemocytes from loss-of-function Rho family GTPases – Cdc42, Rac1, and Rac2 were analysed for any variances in motile behaviour when compared to heterozygous controls of each respective loss-of-function strain. All cells were analysed on a 0.2% gelatine ECM in standard ex vivo conditions. (A) Track plots for heterozygous and homozygous *Cdc42^2^* WPP haemocytes, (B) track plots for heterozygous and homozygous *Rac1^J11^* WPP haemocytes, and (C) track plots for heterozygous and homozygous *Rac2* ^Δ^ WPP haemocytes. Heterozygous controls were created by crossing each loss-of-function strain into a either *w^1118^* (used for *Cdc42^2^* and *Rac1^J11^*) or *ry506* (used for *Rac2* ^Δ^).

White pre-pupal haemocytes from homozygous loss-of-function Rho GTPases – *Cdc42^2^*, *Rac1^J11^* and *Rac2^Δ^*, were tested to see if there was any variation in random motility in comparison to heterozygous controls. This comparison was tested using migration velocity, total distance travelled, and confinement ratio (Figure 4).

*Cdc42^2^* WPP haemocytes were observed to exhibit motile behaviour, but in some cases in an abnormal fashion. The cells were capable of protrusion, similar to that observed in wild-type WPP haemocytes, but the abnormality was a lack of coherent retraction at the posterior end (Film S4). This lead to an elongated cell until the initial protrusion returned to its point of origin. After comparison to heterozygous controls, *Cdc42^2^* vs. *Cdc42^2^*/+, it was determined that migration velocity and total distance travelled were reduced by 63% and 65%, respectively with significance of P<0.001. With regards to the confinement ratio, it was determined that homozygous *Cdc42^2^* exhibited a 56% increase, but this was not significant (P>0.05) (Figure 4A and Table 2).

**Table 2:** Summary of motile characteristics for heterozygous and homozygous loss-of-function Rho family GTPases – Cdc42, Rac1, and Rac2.

Characteristic:	*Cdc42^[2]^*+/− −/−		*Rac1^J11^*+/− −/−		*Rac2* ^Δ^+/− −/−	
**Velocity (µm/s)**	0.55 (±0.04)	0.20 (±0.05)	0.57 (±0.03)	0.19 (±0.02)	0.40 (±0.03)	0.21 (±0.02)
**Distance (µm)**	630 (±41)	216 (±24)	655 (±36)	224 (±23)	450 (±37)	277 (±23)
**Confinement ratio**	0.07 (±0.01)	0.16 (±0.03	0.07 (±0.01	0.11 (±0.02	0.11 (±0.02	0.11 (±0.01

Summary of motile characteristics – migration velocity, total distance travelled and confinement ratio, for each respective heterozygous (control) and homozygous Rho family GTPase loss-of-function strains. Statistical analysis, signified by the line and asterisk above, was performed to determine any significant difference in each motile characteristic between heterozygous control, and homozygous WPP haemocytes on 0.2% gelatine under random *ex vivo* conditions.

Homozygous strains *Rac1^J11^* and *Rac2^Δ^* both appeared less motile than wild-type WPP haemocytes and their respective heterozygous controls (Film S5 and Film S6). There was some minor movement around a fixed axis, similar to non-motile 3^rd^ instar haemocytes, but there were no characteristic signs of WPP haemocyte motility. In both cases, *Rac1^J11^* and *Rac2^Δ^*, WPP haemocytes exhibited significantly decreased, P<0.001, migratory velocities, and total distance travelled (Figure 4B and 4C). For *Rac1^J11^* migration velocity and total distance travelled were reduced by 66% in both respects, whereas for *Rac2^Δ^* migration velocity and total distance travelled were reduced by 47% and 28%, respectively. Finally the confinement ratio was determined for both *Rac1^J11^* and *Rac2^Δ^*, only *Rac1^J11^* showed variation by an increase in confinement ratio by 36%, which showed no significance P>0.05 (Table 2).

## Discussion

The *ex vivo* technique for incubating primary *Drosophila* post-embryonic haemocytes provides an arena for multiple cellular assays. Pioneering this technique, a basic characterisation of circulating haemocyte behaviour in larvae and white pre-pupae was conducted in conjunction with the aim to discover if post-embryonic haemocytes are capable of migration. All of this was carried out in order to produce a standard characterisation of various attributes in haemocyte cytoskeleton dynamics, within a random 2D system, so that this information can be used as a comparison for further research. This study analysed basic haemocyte behaviour, determined by general morphology, protrusion activity and general cytoskeleton dynamic behaviour, across all larval life stages and pre-pupae. Motility in loss-of-function Rho family GTPases- Cdc42, Rac1 and Rac2, was also assessed. Finally, the data produced has created a standardised initial characterisation of haemocyte motility at the pre-pupal life stage.

### Larval haemocyte configuration and behaviour

The results determined that haemocytes from the larval instar stages are generally static, only appearing to have actively protruding plasma-membranes, with non-significant retrograde flows (Figure 1C). Although the protrusion frequency is variable between each life stage, there is a general dormancy amongst larval haemocytes in comparison to pre-pupal haemocytes. *Drosophila* larval stages are known to store energy in preparation for the final stage of morphogenesis [20]. Haemocytes, for example plasmatocytes, have many functions and are assumed to activate when the host is under stress. Therefore, under normal conditions, where no stress is present, this dormant behaviour may be a means to conserve energy [11], [14]. However, there is variance in the behaviour of first instar haemocytes in comparison to haemocytes of the second and third instars. This dynamic behaviour, which is characteristically similar to pre-pupal haemocytes, could be reminiscent of haemocyte behaviour at the embryo stage, whereby haemocytes are more dynamic (greater membrane activity) and motile [1], [4]. It is possible that the first instar haemocyte population consists of both larval and embryonic haemocytes. Embryonic haemocytes are known to actively proliferate at post-embryonic life stages, therefore the behaviour we observed might be due to contaminating embryonic haemocytes [21], [22].

To determine general adhesive and spreading ability, haemocytes from third-instar larvae were subjected to three different ECM substrates. On laminin and fibronectin haemocytes produced specific phenotypes in comparison to haemocytes on glass or gelatine. Haemocytes on laminin formed multiple filopodia, whilst on fibronectin the haemocytes had extremely spread plasma-membranes. Laminin is known to interact with *Drosophila* αPS1βPS,αPS1 is encoded by *multiple edematous wings* (*mew*) in *Drosophila*, which is activated upon interaction with laminin [23]. Whilst fibronectin, although not a naturally produced extra-cellular matrix of *Drosophila*, was shown to interact with αPS2βPS, αPS2 is encoded by *inflated* (*if*) in *Drosophila* [23]. It is not know as to why haemocytes produce multiple filopodia on laminin, nor is it known why haemocytes are extremely spread on fibronectin.

### Post-embryonic haemocyte motility

It was proposed that haemocyte motility only existed at the embryonic stage of *Drosophila* development [1]–[5], [17]. From these pioneering studies it is now evident that haemocyte migration is not limited to the embryonic stage. As yet, it is unknown as to why haemocytes exhibit a non-motile state during the larval instar life stages, but change their behaviour at the pre-pupal stage. As pre-pupation is a prequel to the final step in *Drosophila* morphogenesis it is known that the larvae are preparing for organ development, as well as rearrangement, which ties in with a surge of the ecdysteroid, 20-hydroxyecdysone that induces mass cell death [10], [20]. This in turn could result in activation of motile behaviour in the existing haemocytes, assuming that all haemocytes possess a dormant motile behaviour, or that these haemocytes are life-stage specific. It has been previously observed that ecdysone affects cell adhesion and morphology, as well as induction of mass cell death producing highly active phagocytic cells in the embryonic life stage [1], [10]. It is known that *Drosophila* morphogenesis induces two waves of haemocyte production, an embryonic wave and a larval wave [21]. This suggestion of a tertiary wave cannot be supported by this study, but it is clear that motility is induced in preparation for morphogenesis. Apart from ecdysteroid hormone induced motility, motility could also be observed due to changes in the natural extra-cellular matrices to which the cells adhere. An extra-cellular matrix exhibits a uniform spread of inter-connected ligands [24], [25]. This ‘net of connections’ creates a regulated tensile stress across a surface [26]. Motility is a product of variation in the tensile stress across an ECM, induced by a sudden tear or open division in the uniform surface. As morphogenesis is a period of change, organ development and re-distribution of organs, this could lead to variances in the spread of ECM ligands, thus varying the uniform tensile stress, which could induce migration [27]–[29]. As we are not extracting natural ECM from in vivo, as well as not knowing what ECM these cells adhere to *in vivo*, the use of gelatine in ex vivo could simulate that change uniform tensile stress assuming that path length between ligands is different to the *in vivo* ECM.

Removing Rho-family GTPases – Cdc42, Rac1, or Rac2 provided some evidence as to how WPP haemocyte motility is controlled and possibly driven. Although the *ex vivo* system is totally random, the presence or absence of motility was assessed. Removal of Cdc42 lead to no loss of motility, but caused some haemocytes to act abnormally. Haemocytes lacking Cdc42 activity became elongated upon protrusion at the anterior edge, whilst lacking retraction at the posterior edge. Lack of Cdc42, in a stereotypical model of migration, induces loss of actin polymerisation at the leading edge, therefore reducing filopodia formation [30]–[32], which in our case may have caused the abnormal protrusive nature at the anterior edge. When either Rac1 or Rac2 were removed, haemocytes appeared less motile and exhibited behaviour similar to 3^rd^ instar haemocytes. Whilst Rac1 and Rac2 are also responsible for actin-polymerisation they lead to formation of lamellipodia, therefore loss of either could induce loss of/aberration of lamellipodia formation, leading to a lack of motility [16], [33]. Since cell migration is essentially a ‘walking’ process the cell must create multiple points of contact with an extra-cellular matrix, via integrin-ligand bonds. If you remove the filopodia you are not severely reducing the number of integrin-ligand bonds as overall surface area is not majorly affected, whereas removal of lamellipodia would lead to a major decrease in overall surface area for cell interaction with the substrate therefore reducing the number of integrin-ligand bonds.

The discovery of haemocyte motility in pre-pupae not only highlights the versatility of the *ex vivo* platform, but also opens up new avenues for using *Drosophila* as a standardised model of polarised and motile cell research. The application of this research is currently limited to a 2D model, but as the initial step has been made, there is now a broad spectrum of progressive improvement toward 3D models [34], [35]. Apart from the technique, this study has once again shown that *Drosophila* is a powerful organism that has an exploitable repertoire of systems, which can be used to advance our current knowledge of science as it is presently understood. Furthermore, we propose this *ex vivo* technique could be used to study haemocyte-microbe interactions, for example *Pseudomonas aeruginosa* which is the most clear case of a microbe that suppresses host cell cytoskeleton in *Drosophila* [36], [37].

## Materials and Methods

### Fly stocks

All assays were performed on the lab control strain *w^1118^*, apart from adhesion and spreading assays, which are detailed below. All GAL4-UAS assays used the well described *Hemese-GAL4* (*He-GAL4*) driver [38]. Flies were grown and maintained on a standard cornmeal diet (60% w/v yellow cornmeal, 12% w/v inactive dry yeast, 7.5% v/v golden syrup, 1.4% w/v methyl paraten (tegosept), and 6% w/v agar) at room temperature ∼23–25°C and under naturally regulated light and dark conditions, except for GAL4-UAS strains, which were allowed to mate for 48 hours at room temperature then incubated at 29°C to drive ectopic expression. The various loss-of-function and gain-of-function strains used in the study are as follows:

Various RhoGTPase non-active and constitutive-active strains used:

Rac1 strains:

1. y^1^ w^*^; Rac1^J11^ P{FRT(w^hs^)}2A/TM6B, Tb^1^

Rac2 strains:

1. Rac2^Δ^ ry^506^

Cdc42 strains:

1. y^1^ w^*^ Cdc42^2^ P{neoFRT}19A

### Ex-vivo incubation of isolated haemocytes

A round glass bottom petri-dish, of 35 mm external diameter and 15 mm internal glass diameter, was used as the main chamber for *ex vivo* incubation, these chambers contained a #1 round glass coverslip (MatTek corp.). If desired, 1 hour before the main haemocyte isolation, 100 µl of various extra-cellular matrix (ECM) substrates, for purposes of assays involving ECM, were applied to the sterile glass surface at room temperature. Following ECM application, after 40 minutes through the coating time, 6 larvae were collected and placed into dH_2_O over the remaining 20 minutes, for cleaning. All larval instars were staged using mouth hook morphology, further late third-instar larvae were staged using red food colouring in the food, to differentiate early third-instar from late third-instar larvae. Once cleaned, ensuring all debris was removed from the larval surface, larvae were bled at the dorsal posterior end, between the dorsal trachea, with a sterilised 25 gauge needle, into 100 µl of *Drosophila* haemocyte isolate medium (DHIM). DHIM consists of 75% (v/v) 1× Schneider's *Drosophila* medium (1xSDM) and 25% (v/v) 1× heat-inactivated fetal bovine serum (1xFBS). Immediately after haemocyte isolation, any non-adherent ECM substrate was washed from the glass coverslip. The 100 µl of DHIM, containing isolated haemocytes, was transferred to the coated glass surface and an extra 100 µl of DHIM was used to wash the bleeding spot, in order to uplift any remaining haemocytes. The cells were then incubated at 25°C for 1 hour to allow the cells to spread on the ECM. After 1 hour, 0.3 ml of DHIM was further added to the chamber for excess supplementation of the cells in order to allow for analysis of cell behaviour over greater periods of time. Cells were viewed using phase-contrast microscopy to produce time-lapse AVI files at 15 seconds per frame.

To demonstrate directed cell migration, cells were extracted and incubated as described above. During this incubation time, one of the previously bled white pre-pupae was dissected further to excise a section of the gut (thus rupturing the gut if not done so already), observed as a long greyish-white string under a stereo-light microscope, and then incubated separately in DHIM. This was done parallel to WPP haemocyte incubation for 1 hour at 25°C. Healthy cells were then viewed under time-lapse before addition of the dying tissue, then continuing with time-lapse imaging post dying tissue addition.

### Application of extra-cellular matrices

The application of ECM is so that the cells may behave closer to their *in vivo* environment, and not be influenced by the electrostatic attraction with naked glass. It is understood that any ECM application, at this current stage, will not be in keeping with the wild type standard but this also allows for variation in method, and broadens the application of the *ex vivo* technique. The ECM substrates used in this study were gelatine (digested collagen, stored at room temperature) [MERCK – 1.04080], laminin [Sigma Aldrich – L2020] (stored at 4°C), and fibronectin [Sigma Aldrich – F0895] (stored −20°C). Each substrate was diluted to a standard 1∶40 ratio, apart from gelatine which was used at 0.2% v/v in 1× phosphate buffer saline (1xPBS). Laminin was diluted in laminin buffer (50 mM Tris-HCL, pH 7.5, 150 mM NaCl). After 1 hour coating time, the substrate was removed. In the cases of laminin and fibronectin there were two washes with 1xPBS after ECM removal, in order to eliminate the chemicals within the substrate solutions. For purposes of this study, the preferred ECM used was gelatine as cells behaved normally on a regular basis, whereas on laminin the cells were erratic in morphology and behaviour, and with regards to fibronectin it is known that *Drosophila* does not produce fibronectin naturally therefore use of this ECM has been retained to ECM specific studies.

### Imaging

Imaging was performed on an inverted Zeiss Axiovert 200 M series automated microscope. Cellular detail was provided by the use of a Zeiss 63× oil-immersive objective with numerical aperture of 1.4, set to phase contrast 3 (Ph3), and captured using an ORCA-ER C4742-80 digital camera controlled by simplePCI imaging software. Filter, and condenser, set-up was set to Ph3 in order to coincide with the objective being used and also the condenser was set to its most dilated state. All data was collected as a time-lapse collage, which was then condensed into an AVI movie file.

### ImageJ: Setting up ImageJ

ImageJ v1.41 (NIH), was used to analysis of all AVI files collected from the *ex vivo* technique. In order to analyse the data effectively ImageJ was used to read each AVI in terms of microns rather than pixels. Upon opening ImageJ and loading the desired AVI, the ‘straight line ROI’ was selected. A single straight line was drawn equalling a length of 100 pixels. Since we used a 63× objective, the known distance of 100 pixels in microns is 1.6 µm with a pixel aspect ratio of 63 (1 µm = 63 pixels). ‘Analyze’>‘set scale’ was then selected, and the above parameters were set. The unit of length to be measured was labelled as ‘microns’ and global distribution was ticked. This was the basic set-up before any analysis can be conducted.

### Kymograph and retrograde flow

Each cell of interest was marked, labelled (to ensure no same cell was measured twice) and individually analysed. Firstly, the chosen cell was magnified to 150%. After this a line was drawn from just within the centroid region to the exterior of the plasma-membrane (PM). The clearance of the line from the PM was enough to ensure that the PM did not extend over the ROI line drawn. A kymograph was then produced from this straight line, with a frame rate of 10 seconds. On the kymograph there were minor regions of phase dense and phase sparse striations. Straight ROI line was used further, and a line was drawn along the length of each individual striation, whether phase dense or sparse. The ROI line needed to stay within the PM region so that retrograde flow was not confused with the lamella flow. Retrograde flow refers the rate of retraction against the rate of protrusion at the plasma-membrane edge, which gives definition to the micro-tubule rate of polymerisation and thus extension, when a cell is exhibiting protrusive behaviour. Once the line was drawn, an ImageJ plugin ‘read velocity tsp’ was selected, and the velocity of the retrograde flow appeared in a separate window. Overall there were at least 8 kymographs produced per cell, with a minimum of 8 striations per kymograph analysed.

### Protrusion frequency

Protrusion frequency is a measurement of the amount of protrusions per second, within a fixed time frame and provides an idea of general protrusive activity. Kymographs from each cell, within each respective variant, were collected and then analysed for how many protrusions were present within a constant time, which in our case was ten seconds. Each peak within a kymograph was only analysed if it fell into the following set of rules:

1. Each protrusion should originate from the plasma-membrane region, not the centroid region.
2. Each kymograph is a product of a 10 second time slice.
3. Each protrusion must have a minimum length (amplitude) of 30 pixels, showing a definitive trough.
4. Any bi- and tri- peaks will be counted as a single peak within one protrusion.

Once all protrusions had been summarised, the following formula was applied to determine the protrusion frequency: f = N/T, where f = Protrusion frequency (protrusions/second), N = Number of peaks (protrusions) in the kymograph and T = Time (seconds).

### Characterisation of cell motility

Cell motility was characterized within five parameters of motile cellular behaviour - velocity, arrestment coefficient, confinement ratio, turning angle, and directionality. The first piece of data collected was a track plot of all migrating cells. This was done using ImageJ plug-ins ‘MTrackJ’ or ‘Manual tracking’ Tracking parameters were set to the following: time intervals: 15 seconds, distance calibration: 1.6 µm, dot size: 5.0 and line width: 1.0. The cell was followed until all frames had passed. If more tracks were to be made then ‘add tracks’ was clicked again and the above was repeated until all cell tracks were made. Once all tracks were completed a results window showed a summary of all x and y co-ordinates collected. Now using a stand-a-lone program ‘chemotaxis and migration tool’ (Ibidi, cells in focus, written by Gerhard Trapp) the spread sheet was loaded up. In order to view the track plot, the data set is first ‘initialised’ under these settings: Number of slices: ‘Use slices from … to …’ (first frame to last frame.), X/Y calibration: 1.6 µm and Time interval: 15 seconds. 

### Calculating migration velocity and arrestment coefficient

Migration velocity refers the speed over a particular displacement, within an observed vector, whereas the arrestment coefficient refers to the amount of time when a cell remains on a fixed spot during the path of migration, also presented in units of velocity. Both migration velocity and arrestment coefficient were calculated automatically through the stand alone program ‘chemotaxis and migration tool’ (Ibidi, written by Gerhard Trapp) once all cells had been track plotted.

### Mean displacement and confinement ratio

The mean displacement, determined as the shortest distance between two points (start and end), gives an indication of the readily able the cell is to move along a random path of migration. This also aids in determining the confinement ratio, a ratio of the total path length over the mean displacement, showing how confined a cell migrates around a fixed position. Mean displacement, and total path length were calculated automatically by the program ‘chemotaxis and migration tool’ and the confinement ratio was determined as a division of mean displacement over total path length. Once all individual displacements had been gathered, this data was placed into an excel spread sheet where the mean was calculated with the standard error.

### Turning angle

The turning angle is used to describe the external angle of a turn within 360° when the cell is changing direction during its path of migration, and also shows the number of turns taken during migration. The turning angle was measured from the migration track of a cell in ImageJ. After the cells path of migration had been manually tracked, using plugin ‘MTrackJ’, each turning point in the track was marked, and labelled, then straight arrowed lines were drawn between each turning point. Each arrow assumed a particular trajectory and then the external angle of the turn made, from the original trajectory to the new trajectory, was measured. All turning angles, as well as the number of turning points, were summarised and then the mean, and the range, of the turning angle were determined.

### Directionality

Directionality is a reference to how direct a cell is toward a target along a random migratory walk. Directionality was calculated automatically from the program ‘chemotaxis and migration tool’. All directionality data was accumulated and placed into an excel spreadsheet to determine the mean and standard error.

### Statistics

Haemocyte behaviour at the different life stages and on different ECM substrates was confirmed from a minimum of 32 larvae, which provided the same observations in a minimum of 3 repeat experiments. In these assays the total number of haemocytes, for each respective variant, was summed together as a total haemocyte number then the observed phenotype (in a number of haemocytes) was given a percentage of the total sample population. For the migration analysis, for each respective motile characteristic, a one sample Student's t-test was used, driven by the statistics program SPSS v18. Haemocyte motility analysis also used a minimum of 32 cells to produce all data, for the given criteria, in the form of a data range, mean and the standard error.

## Supporting Information

Film S1 **Time lapse of a non-polarised white pre-pupal haemocyte on 0.2% gelatine extra-cellular matrix.** Time lapse imaging was performed on primary haemocytes, excised from the white pre-pupal life stage of *w^1118^ D. melanogaster*, in *ex vivo* conditions on a 0.2% gelatine ECM. The haemocyte has been cropped to 150% greater than its original, and has been rendered from an 80 frame time lapse, which was taken with a frame rate of 15 seconds. The haemocyte visualised is exhibiting what was classed as a ‘non-polarised’ phenotype, which was one of six common phenotypes observed.
(AVI)

Film S2 **Time lapse of a polarised white pre-pupal haemocyte on 0.2% gelatine extra-cellular matrix.** Time lapse imaging was performed on primary haemocytes, excised from the white pre-pupal life stage of *w^1118^ D. melanogaster*, in *ex vivo* conditions on a 0.2% gelatine ECM. The haemocyte has been cropped to 150% greater than its original size, and has been rendered from an 80 frame time lapse, which was taken with a frame rate of 15 seconds. The haemocyte visualised is exhibiting what was classed as a ‘polarised’ phenotype, which was one of six common phenotypes observed.
(AVI)

Film S3 **Time lapse of directed migration of white pre-pupal haemocytes on 0.2% gelatine extra-cellular matrix, toward dead tissue debris.** Time lapse imaging was performed on primary haemocytes, excised from the white pre-pupal life stage of *w^1118^ D. melanogaster*, in *ex vivo* conditions on a 0.2% gelatine ECM. The time lapse has been rendered from an 80 frame time lapse, which was taken with a frame rate of 15 seconds. The haemocytes visualised are demonstrating contact initiated directed cell migration toward a target of dying tissue debris that was previously exuded from a dissected section of gut, which was left to decay separately in *ex vivo* before its addition to the healthy system.
(AVI)

Film S4 **Time lapse of a** ***Cdc42^[2]^*** **white pre-pupal haemocyte on 0.2% gelatine extra-cellular matrix.** Time lapse imaging was performed on primary haemocytes, excised from the white pre-pupal life stage of *Cdc42^[2]^* *D. melanogaster*, in *ex vivo* conditions on a 0.2% gelatine ECM. The haemocyte has been cropped to 150% greater than its original, and has been rendered from an 80 frame time lapse, which was taken with a frame rate of 15 seconds. The haemocyte was observed to protrude abnormally, with no coherent posterior retraction.
(AVI)

Film S5 **Time lapse of a** ***Rac1^J11^*** **white pre-pupal haemocyte on 0.2% gelatine extra-cellular matrix.** Time lapse imaging was performed on primary haemocytes, excised from the white pre-pupal life stage of *Rac1^J11^ D. melanogaster*, in *ex vivo* conditions on a 0.2% gelatine ECM. The haemocyte has been cropped to 150% greater than its original, and has been rendered from an 80 frame time lapse, which was taken with a frame rate of 15 seconds. The haemocyte was observed to produce the active non-polarised phenotype, and showed some minor movement around a fixed axis.
(AVI)

Film S6 **Time lapse of a Rac2^Δ^ white pre-pupal haemocyte on 0.2% gelatine extra-cellular matrix.** Time lapse imaging was performed on primary haemocytes, excised from the white pre-pupal life stage of *Rac2^Δ^ D. melanogaster*, in *ex vivo* conditions on a 0.2% gelatine ECM. The haemocyte has been cropped to 150% greater than its original, and has been rendered from an 80 frame time lapse, which was taken with a frame rate of 15 seconds. The haemocyte was observed to produce a non-polarised phenotype with a plasma-membrane spread in close proximity to the centroid region. There was some minor movement but only around a fixed axis.
(AVI)

## References

[1] Tepass U, Fessler LI, Aziz A, Hartenstein V (1994). Embryonic origin of haemocytes and their relationship to cell death in *Drosophila*.. Development.

[2] McDonald JA, Montell DJ (2005). Analysis of cell migration Using *Drosophila* as a model system.. Methods Mol Biol.

[3] Paladi M, Tepass U (2004). Function of Rho GTPases in embryonic blood cell migration in *Drosophila*.. J Cell Sci.

[4] Wood W, Faria C, Jacinto A (2006). Distinct mechanisms regulate hemocyte chemotaxis during development and wound healing in *Drosophila* melanogastor.. J Cell Biol.

[5] Wood W, Jacinto A (2007). *Drosophila melanogastor* embryonic haemocytes: masters of multitasking.. Nat Rev Cell Biol.

[6] Irving P, Ubeda J-M, Doucet D, Troxler L, Lagueux M (2005). Ninsights into *Drosophila* larvae haemocyte functions through genome-wide analysis.. Cell Microbiol.

[7] Markus R, Laurinyecz B, Kurucz E, Honti V, Bajusz I (2009). Sessile hemocytes as a hematopoietic compartment in *Drosophila melanogastor*.. Proc Natl Sci Acad Sci U S A.

[8] Lemaitre B, Hoffmann J (2007). The host defence of *Drosophila melanogastor*.. Ann Rev Immun.

[9] Labrosse C, Eslin P, Doury G, Drezen JM, Porie M (2005). Haemocyte changes in *D. melanogastor* in response to long gland components of the parasitoid wasp *Leptopilina boulardi* a RhoGAP protein as an important factor.. J Ins Phys.

[10] Lanot R, Zachary D, Holder F, Meister M (2001). Postembryonic haematopoiesis in *Drosophila*.. Dev Biol.

[11] Crozatier M, Meister M (2007). *Drosophila* haematopoiesis.. Cell Microbiol.

[12] Bidla G, Lindgren M, Theopold U, Dushay MS (2005). Hemolymph coagulation and phenoloxidase in *Drosophila* larvae.. Dev Comp Immun.

[13] Bidla G, Dushay MS, Theopold U (2007). Crystal cell rupture after injury in *Drosophila* requires the JNK pathway, small GTPases and the TNF homolog Eiger.. J Cell Sci.

[14] Meister M (2004). Blood cells of *Drosophila*: cell lineages and role in host defence.. Curr Opin Immun.

[15] Williams MJ, Ando I, Hultmark D (2005). *Drosophila melanogastor* Rac2 is necessary for a proper cellular immune response.. Genes Cells.

[16] Williams MJ, Habayeb MS, Hultmark D (2007). Reciprocal regulation of Rac1 and Rho1 in *Drosophila* circulating immune surveillance cells.. J Cell Sci.

[17] Waddell S, Quinn WG (2001). What can we teach *Drosophila*? What can they teach us?. Trends Genetics.

[18] Cox EA, Huttenlocher A (1998). Regulation of Integrin-Mediated Adhesion During Cell Migration.. Mic Res Tech.

[19] Bretscher MS (2008). On the shape of migrating cells – a ‘front-to-back’ model.. J Cell Sci.

[20] Dubrovsky EB (2005). Hormonal cross talk in insect development.. Trends Endocrin Metab.

[21] Holz A, Bossinger B, Strasser T, Janning W, Klapper R (2003). The two origins of haemocytes in *Drosophila*.. Development.

[22] Truman JW, Riddiford LM (1999). The origins of insect metamorphosis.. Nature.

[23] Dinkins MB, Fratto VM, LeMosy EK (2008). Integrin alpha chains exhibit distinct temporal and spatial localization patterns in epithelial cells of *Drosophila* ovary.. Dev Dyn.

[24] Boisvert M, Chetoui N, Gendron S, Aoudjit F (2010). Alpha2beta1 integrin is the major collagen-binding integrin expressed on human Th17 cells.. Eur J Immunol.

[25] Baek SH, Kwan YC, Lee H, Choe KM (2010). Rho-family small GTPases are required for cell polarisation and directional sensing in *Drosophila* wound healing.. Biochem Biophys Res Comm.

[26] Huttenlocher A, Sandborg RR, Horwitz AF (1995). Adhesion in cell migration.. Curr Opin Cell Biol.

[27] Fukata M, Nakagawa M, Kaibudi K (2003). Roles of Rho-family GTPases in cell polarisation and directional migration.. Curr Opin Cell Biol.

[28] Huveneers S, Danen EHJ (2009). Adhesion signalling-crosstalk between integrins, Src and Rho.. J Cell Sci.

[29] Pfandtner J, Lyman E, Pollard T, Voth G (2010). Structure and dynamics of the actin filament.. J Mol Biol.

[30] Szczur K, Xu H, Atkinson S, Zheng Y, Filipi M-D (2006). Rho GTPase CDC42 regulates directionality and random movement via distinct MAPK pathways in neutrophils.. Blood.

[31] Szczur K, Zheng Y, Filipi M (2009). The small Rho GTPase Cdc42 regulates neutrophil CD11b integrin signalling.. Blood.

[32] Heasman SJ, Ridley AJ (2008). Mammalian Rho GTPases: new insights into their functions from in vivo studies.. Nat Rev.

[33] Songklanakarin J, Wanichpakorn S, Kedjarune-Laggat U (2010). Primary cell culture from human oral tissue: gingival keratinocytes, gingival fibroblasts and periodontal ligament fibroblasts.. Sci Technol.

[34] Friedl P, Bröcker EB (2000). The biology of cell locomotion within three-dimensional extracellular matrix.. Cell Mol Life Sci.

[35] Grinnell F, Petroll WM (2010). Cell motility and mechanics in three-dimensional collagen matrices.. Ann Rev Cell Dev Biol.

[36] Avet-Rochex A, Bergeret E, Attree I, Meister M, Fauvarque MO (2005). Suppression of *Drosophila* cellular immunity by directed expression of the ExoS toxin GAP domain of Pseudomonas aeruginosa.. Cell Microbiol.

[37] Apidianakis Y, Mindrinos MN, Xiao W, Tegos GP, Papisov MI (2007). Involvement of skeletal muscle gene regulatory network in susceptibility to wound infection following trauma.. PLoS One.

[38] Kurucz E, Zetterval CJ, Sinka R, Vilmos P, Pivarcsi A (2003). Hemese, a haemocyte-specific, transmembrane protein, affects the cellular immune response in *Drosophila*.. Proc Natl Sci Acad Sci U S A.

